# The role of perceived competence in remote cochlear implant aftercare: a mixed-methods study

**DOI:** 10.3389/fdgth.2026.1803067

**Published:** 2026-06-03

**Authors:** Maika Werminghaus, Susann Thyson, Nora Sieverding, Emily Breitenbach, Jutta G. Richter, Thomas Klenzner

**Affiliations:** 1 Department of Otorhinolaryngology/Cochlear Implant Center, Medical Faculty and University Hospital Duesseldorf, Heinrich-Heine-University Duesseldorf, Duesseldorf, Germany; 2 Department of Rehabilitation & Special Education, Faculty of Human Sciences, University of Cologne, Cologne, Germany; 3 Department of Rheumatology, Medical Faculty and University Hospital Duesseldorf, Heinrich-Heine-University Duesseldorf, Duesseldorf, Germany; 4 Hiller Research Center, University Hospital Duesseldorf, Medical Faculty and University Hospital Duesseldorf, Heinrich-Heine-University Duesseldorf, Duesseldorf, Germany; 5 Center for Digital Medicine, University Hospital Duesseldorf, Medical Faculty and University Hospital Duesseldorf, Heinrich-Heine-University Duesseldorf, Duesseldorf, Germany

**Keywords:** cochlear implantation, digital health, health literacy, mixed methods, patient acceptance of health care, remote care, self-determination-theory, telemedicine

## Abstract

**Introduction:**

Remote care and digital health tools are increasingly incorporated into cochlear implant aftercare to enhance accessibility and patient engagement. Their uptake, however, depends strongly on perceived competence, digital health literacy, and motivational factors among patients with cochlear implants (CI).

**Methods:**

This exploratory sequential mixed-methods study investigated motivational mechanisms and digital readiness among patients with cochlear implants (PwCI). In the qualitative phase, three semi-structured group interviews (*n* = 9) explored motivational drivers, barriers, and acceptance conditions. In the quantitative phase, 62 PwCI completed standardized instruments assessing affinity for technology, digital health literacy, and motivation for technology adoption.

**Results:**

Qualitative findings highlighted autonomy-related benefits such as flexibility and time savings, alongside competence-related concerns including technical uncertainty and the need for professional reassurance. Quantitative analyses showed moderate to high levels of digital health literacy and technology affinity, with perceived competence strongly associated with self-determined motivation and related engagement intentions. Technology affinity emerged as the strongest predictor of perceived competence.

**Discussion:**

Engagement with remote CI aftercare appears to depend less on technical availability alone than on perceived competence and motivational factors, underscoring the importance of competence-supportive design and hybrid care models in digital aftercare implementation.

## 1 Introduction

In light of demographic change, increasing case numbers, and workforce constraints, remote care has gained momentum as a complement to conventional, in-person cochlear implant (CI) aftercare (1–3). Given that CI aftercare represents a continuous, lifelong process, remote service models may complement conventional on-site follow-up and help address resource constraints. In the context of CI rehabilitation, remote care refers to the digitally supported monitoring, assessment, and partial management of CI systems and rehabilitation processes without requiring the patient's physical presence at the outpatient clinic. It is based on bidirectional data exchange between the CI processor, personal devices (e.g., smartphones or tablets), and clinical infrastructures, typically mediated via online interfaces that are designed to meet data protection and security requirements (4). All major CI manufacturers provide remote-care features within their proprietary ecosystems, such as CochlearTM Remote Check, MED-EL HearCare, or AB Remote Support (Advanced Bionics), offering functionalities like device status monitoring, data transmission, and remote or asynchronous review of sessions by clinical staff. Patients with cochlear implants (PwCI) can monitor device use, perform remote self-tests, complete digital questionnaires, and even conduct basic hearing or speech tests independently, whereas complex device programming and clinical decision-making currently remain the responsibility of clinical professionals and are predominantly performed during in-clinic appointments (4, 5).

Beyond device management, digital hearing-training programs (dHTP) represent another emerging form of remote rehabilitation, enabling auditory training and progress tracking outside clinical environments (3, 6, 7). Recent dHTP concepts increasingly focus on adaptive difficulty, user-centered feedback, and individualized training trajectories (8). Such programs provide structured, home-based auditory exercises extending rehabilitation beyond the clinic. Their relevance extends beyond training itself, as dHTP usage requires sustained engagement, basic digital skills, and autonomous navigation of digital interfaces, making dHTP a central context for understanding user readiness for remote-care solutions.

Within the broader eHealth landscape, remote care in CI rehabilitation aims to enhance accessibility, efficiency, and patient empowerment across the lifelong trajectory of CI use (8–13). A systematic review published nearly a decade ago already demonstrated that remote CI programming can achieve outcomes comparable to in-person fitting and is well accepted by patients, underscoring the early feasibility of remote-care approaches (14). By integrating real-world data collection and self-management features, remote-care systems may reduce clinical workload, foster autonomy, and support more personalized, adaptive follow-up (15). Scientific evaluations demonstrated equivalence to in-person appointments and high satisfaction among PwCI and clinical staff (16–18), while challenges remain concerning technical infrastructure and digital competencies among both patients and professionals (19, 20). Recent studies also showed the validity and stability of self-administered smartphone-based assessments and the real-world feasibility of vendor telehealth tools such as Cochlear TM Remote Check (5, 12, 13).

Despite increasing feasibility and technological maturity, the successful adoption and sustained use of remote care depend strongly on users’ motivational readiness and digital competence, factors that remain underexplored in CI research. While usability problems, uncertainty about technical handling, and hesitancy toward reduced face-to-face contact have been reported, the psychological mechanisms shaping engagement have received limited attention (21, 22). Self-Determination Theory (SDT) offers a robust framework for analyzing these mechanisms by distinguishing intrinsic, extrinsic, and amotivated (a lack of intention or perceived relevance) regulation types and conceptualizing motivation along a continuum from externally controlled to self-determined forms (23–25). SDT emphasizes the satisfaction of three basic psychological needs (autonomy, competence, and relatedness) as central to fostering internalization, performance, and well-being. In health contexts, supporting the internalization of health-related values can enhance self-determined motivation and promote the uptake of health-supportive behaviors, such as hearing aid use (26, 27).

In the context of remote CI aftercare, engagement requires patients to initiate and sustain technology-based health behaviors that are largely self-directed and integrated into everyday life. Such behaviors are not solely determined by technical accessibility, but critically depend on motivational processes and the extent to which they are internalized.

Self-Determination Theory (SDT) provides a particularly suitable framework for this context, as it explains how health-related behaviors are initiated, internalized, and maintained over time. In contrast to technology acceptance models such as the Technology Acceptance Model (TAM) and the Unified Theory of Acceptance and Use of Technology (UTAUT) (28, 29) which primarily focus on initial adoption and perceived usefulness, SDT offers a more comprehensive framework for understanding the motivational processes underlying sustained and self-determined engagement with remote care.

Within this framework, perceived competence is of central relevance, as it reflects individuals’ confidence in effectively using digital health technologies and managing their own care. In remote CI aftercare, where patients are required to independently operate digital systems and interpret feedback, perceived competence can be understood as a key prerequisite for sustained engagement.

Applying SDT to remote CI aftercare provides a theoretical lens for understanding how autonomy-supportive design, competence-enhancing structures, and professional connectedness influence engagement with digital rehabilitation tools. Preliminary findings suggest that remote-care systems may foster autonomy and competence through features such as self-administered hearing tests, feedback, and progress visualization (30). Following the approach of Henshaw et al. (31), who applied SDT to understand motivational processes in auditory training, the present study adopts SDT as its theoretical framework to examine how autonomy, competence, and relatedness shape PwCI's engagement with remote care within an exploratory mixed-methods design.

While previous CI telehealth research has primarily focused on feasibility, clinical equivalence, and satisfaction, few studies have integrated qualitative user perspectives with quantitative indices of motivation and digital competencies of PwCI (2, 32). To address this gap, the present exploratory-sequential mixed-methods study examined motivational and competency-related factors influencing the adoption of remote-care solutions for PwCI in CI aftercare. Qualitative objectives were to explore how PwCI understand and envision remote-care concepts, identifying motivational drivers, barriers, and contextual conditions for acceptance. Quantitative objectives were to assess relationships between self-determined motivation according to SDT, perceived competence, technology affinity, and digital health literacy, and to examine how these factors contribute to confidence and intention to use remote-care tools.

Accordingly, the study addressed the following research questions (RQ):

RQ1: How do PwCI perceive the motivational drivers, barriers and facilitators, and contextual conditions influencing their engagement in remote CI aftercare?
RQ2: What motivational patterns according to SDT (e.g., self-determined vs. externally regulated) emerge from PwCI's experiences with remote care?
RQ3: How do perceived digital competence, digital health literacy, and technology affinity relate to PwCI’s motivation and confidence in using remote-care tools?
  RQ3.1: Does perceived digital competence correlate with digital readiness (digital health literacy and technology affinity)?
  RQ3.2: How strongly are self-determined and externally controlled motivation associated with perceived competence for remote care?
  RQ3.3: Which individual characteristics can predict perceived competence for remote care?

## 2 Materials and methods

### 2.1 Study design

An exploratory-sequential mixed-methods design was applied (33). The study first employed qualitative semi-structured interviews to explore patient perspectives on remote CI aftercare. The findings informed the subsequent quantitative phase and guided the selection of psychometric measures*.* In a subsequent phase, a quantitative online survey was conducted to support and extend the qualitative findings by assessing motivation, technology affinity, and digital health literacy with standardized instruments. This sequential approach allowed for a systematic integration of qualitative and quantitative findings, whereby insights from the qualitative phase informed both the design and the interpretation of the quantitative results.

### 2.2 Sample and setting

Participants were adult PwCI undergoing outpatient rehabilitation. Inclusion criteria were age ≥18 or older, unilateral or bilateral CI provision, sufficient language proficiency to participate in interviews and questionnaires, and written informed consent. Exclusion criteria included severe cognitive impairment, incomplete or invalid data, and minimal daily CI use (<5 h per day, based on objective device usage data routinely recorded by the CI system and reviewed as part of standard CI aftercare). Participants were consecutively recruited over a three-month study period, and all eligible PwCI were invited to participate. All participants were recruited at a large tertiary CI center and took part either in the quantitative or both quantitative and qualitative study phases. Participants included in the qualitative subsample were recruited pragmatically based on their availability during the data collection period and their willingness to participate in group interviews. This approach allowed for the inclusion of diverse perspectives within routine CI aftercare.

### 2.3 Qualitative interviews

A total of three semi-structured group interviews were conducted with nine PwCI, each lasting approximately 60 min and involving two to four participants. The interview sample comprised four participants with single-sided deafness (SSD group), three with bimodal provision (bimodal group), and two with bilateral implantation (bilateral group, see Supplementary Material). Semi-structured interviews were chosen to allow for the exploration of participants’ individual experiences while ensuring comparability across interviews and groups (34). Given that the study involved three interview groups, a structured approach was necessary to ensure that key thematic areas were addressed consistently across all discussions.

In contrast to fully open-ended formats, which may limit comparability, and fully structured formats, which reduce flexibility and interaction, the semi-structured approach enabled a balance between guidance and openness. This was particularly important to maintain a clear distinction between the qualitative and quantitative components of the study and to allow participants to introduce additional perspectives beyond predefined questions. The semi-structured format also supported moderated group discussions by facilitating balanced participation and reducing the risk of individual participants dominating the conversation.

A semi-structured interview guide was used to explore motivational drivers, perceived barriers and benefits, and contextual conditions for remote-care engagement (RQ1). The group-interview format was chosen to stimulate interaction and to elicit both shared and divergent user experiences. Interviews were conducted in a quiet setting and audio-recorded with PwCI's written consent. Transcripts were anonymized and analyzed by means of thematic qualitative content analysis according to Kuckartz (35) using the transcription software MAXQDA 24 (VERBI GmbH, Berlin, Germany). An initial category system was developed deductively based on the research questions and theoretical frameworks (e.g., Self-Determination Theory, digital health literacy, technology affinity). During the coding process, inductive subcategories were created to capture themes emerging directly from the data. Coding was conducted independently by two researchers, with discrepancies resolved through discussion until consensus was reached.

### 2.4 Quantitative surveys

The quantitative component complemented the qualitative findings by assessing motivational patterns and user-related factors relevant for remote-care engagement. To address RQ2 and RQ3, PwCI completed a digital questionnaire implemented in LimeSurvey (LimeSurvey GmbH, Hamburg, Germany) hosted on a secure institutional server. Data were collected anonymously via the online survey platform, while pseudonymized identifiers were used locally at the clinic to enable data management and analysis. The survey comprised standardized instruments selected to assess motivational patterns, perceived digital competence, and user-related factors relevant for remote-care engagement, as outlined below.

Autonomy and Competence in Technology Adoption (ACTA). The ACTA scale (36) assesses self-determined and externally regulated motivation as well as perceived competence in adopting digital technologies. In the context of remote CI aftercare, self-determined use may reflect personally meaningful goals, whereas external demands (e.g., travel distance, time constraints, or clinic capacity) may lead to more externally regulated engagement. ACTA data were used to answer RQ2 by identifying motivational patterns. Perceived competence (ACTA competence subscale) served as the central outcome for RQ3.

Affinity for Technology Interaction (ATI). The ATI scale (37) captures an individual's tendency to actively, openly, and confidently engage with new technical systems. Technology affinity represents a user-side resource that may facilitate digital interaction and support engagement with remote-care tools. ATI scores were used to address RQ3.

Digital Health Literacy (eHEALS). The most widely used instrument to assess digital health literacy is the eHealth Literacy Scale (eHEALS), a self-reported measure that evaluates six core dimensions and is available in various languages (38, 39). It captures individuals’ ability to find, understand, and critically evaluate online health information. In this study, the German revised version of the scale (GR-eHEALS) was used (40). Higher levels of digital health literacy have been linked to more effective health behavior and greater well-being (41). eHEALS scores were used to address RQ3.

In addition to the standardized instruments, the survey included custom items assessing participants’ awareness of remote-care services, previous use, and preferences for different aftercare formats. These items contextualized motivational patterns and provided descriptive insights into user readiness for remote-care engagement.

### 2.5 Data analysis

Quantitative data were analyzed using Microsoft Excel and R Studio (version R-4.4.3). Descriptive statistics were calculated according to the scoring guidelines of each applied scale. Normality was assessed using the Shapiro–Wilk test. Paired-samples t-tests were conducted to compare motivational subscales, and correlations were computed using Spearman's rank coefficients (*ρ*), with effect sizes interpreted according to Cohen (42). For RQ3.3, a multiple linear regression was performed with perceived competence for remote care (ACTA competence subscale) as the dependent variable and technology affinity, digital health literacy, age, and gender as predictors. Before modeling, assumptions of linearity, homoscedasticity, and multicollinearity were examined, with variance inflation factors used to assess multicollinearity. Statistical significance was set at *p* < .05.

### 2.6 Ethics statement

The study involving human participants was reviewed and approved by the Ethics Committee of the Medical Faculty of Heinrich Heine University Düsseldorf (approval number: 2023_2411). All participants provided written informed consent prior to participation. The study was conducted in accordance with the Declaration of Helsinki.

## 3 Results

**Figure 1:**
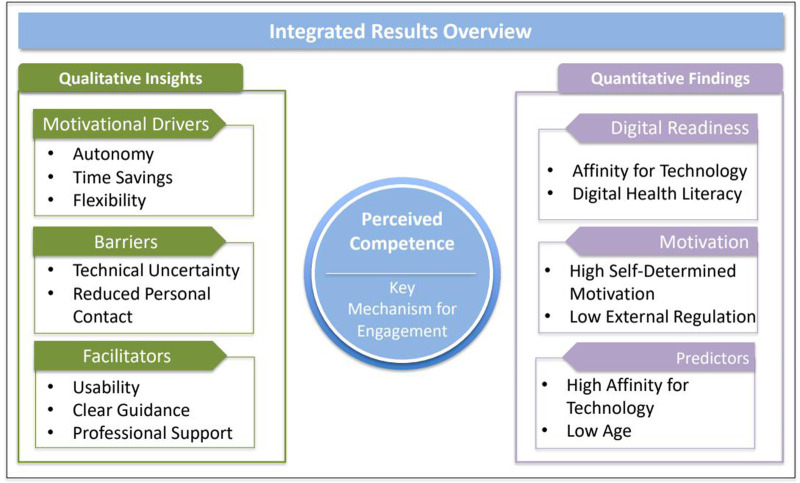
Integrated qualitative and quantitative findings on engagement in remote cochlear implant aftercare. Qualitative insights from group interviews (left) illustrate motivational drivers, barriers, and facilitators shaping engagement. Quantitative survey findings (right) summarize digital readiness factors, including technology affinity, digital health literacy, and motivational regulation. Perceived competence emerged as a central factor associated with both qualitative and quantitative findings related to engagement in remote cochlear implant aftercare.

Results are presented in a structured manner, beginning with sample characteristics, followed by qualitative findings and quantitative analyses. Figure 1 provides an integrated overview of the qualitative and quantitative findings, illustrating perceived competence as a central factor associated with engagement in remote CI aftercare and its relationship with motivational drivers, barriers, facilitators, and digital readiness factors.

### 3.1 Sample characteristics

A total of 72 PwCI were recruited. After data screening and consultation with the supervising audiologists, ten PwCI were excluded due to withdrawal (n = 2), limited CI use, or incomplete questionnaire data (n = 8). The final sample comprised 62 PwCI (28 female, 34 male) aged 18–85 years (M = 56, SD = 15). Participants included 20 users with single-sided deafness (SSD), 27 bimodally fitted users, 13 bilaterally implanted users, and two with unilateral implantation and residual hearing in the contralateral ear. Average daily CI use was 12 h (SD = 3, range = 5–17 h), and mean CI experience was 36 months (SD = 53, range = 1–247 months). Further demographic and clinical characteristics are summarized in Table 1.

**Table 1:** Patients’ demographic data (*N* = 62).

Variable	Value
Age, years, M (SD, Range)	56 (15, 18–85)
Female, % (*n*)	45 (28)
Average daily CI use (hours), M (SD, range)	12 (3, 5–17)
Bilateral hearing with CI systems, % (*n*)	21 (13)
Bimodal provision (hearing aid+CI), % (*n*)	44 + 3* (27 + 2*)
Single-sided deafness with CI, % (*n*)	32 (20)
Duration of hearing with CI, months, M (SD, range)	36 (53, 1–247)
CI Manufacturer, % (*n*)	
Cochlear Ltd.	55 (34)
MED-EL	32 (20)
Advanced Bionics	13 (8)
Native Language, % (*n*)	
German	94 (58)
Arabic, English, Dutch, Portuguese (each *n* = 1)	5 (4)
Highest education level, % (*n*)	
Secondary school certificate or lower	47 (29)
High-school diploma	19 (12)
University degree or postgraduate degree	32 (20)
No formal school-leaving certificate	2 (1)

* PwCI with hearing loss on contralateral ear but no hearing device. Educational levels are based on the German school system and were aligned to internationally comparable categories. Percentages may not total 100 due to rounding.

Of the total sample, nine PwCI participated in three semi-structured interviews as part of the qualitative phase, representing a nested subsample of the overall cohort. PwCI in the interview group were aged 26–72 years (M = 58, SD = 16), with an average daily CI use of 11 h (SD = 2, range = 5–14 h) and a mean CI experience of 29 months (SD = 39, range = 2–138 months).

### 3.2 Qualitative results

#### 3.2.1 Motivational drivers, barriers, and contextual conditions for remote CI aftercare (RQ1)

The qualitative content analysis of the three semi-structured group interviews resulted in four main categories with a total of 13 subcategories (Table 2). None of the PwCI had previously used remote care as part of their CI aftercare, as such services were not available at the study site during the study period. Representative quotations were selected to illustrate central findings and ensure transparency (Supplementary Material). The interviews lasted, on average, 61.3 min (range 57–66 min).

**Table 2:** Main categories of group interviews, subcategories and number of coded segments (N).

Main Category	Subcategory	N (%)
Remote care	Advantages, Disadvantages, Personal Preferences, Financing	29 (76)
Technology affinity	Practical Handling, Digital Competence, Prior Experience with dHTPs	24 (62)
Motivation	Personal Motives, Usage Period	9 (24)
User Experience	Content and Usability Requirements, Feedback and Motivation, Notification, Warning System, Communication and Contact options	38 (100)
Total	­–	100 (262)

3 group interviews, *n* = 9; values represent the number and percentage of coded segments across all interviews (*N* = 262). dHTP, digital Hearing Training Programs.

In line with RQ1, analysis revealed three overarching thematic dimensions relevant for remote-care engagement:

- Motivational drivers: convenience, time savings, and increased autonomy are key reasons for engaging in remote care.
- Barriers and facilitators for use: technical uncertainty, limited digital confidence, and fears of reduced professional contact.
- Contextual conditions for engagement: user-friendly design (e.g., intuitive navigation and clear feedback), clear guidance, and sustained professional support

Across interviews, participants frequently referred to their experiences with dHTP, reflecting the prominent role these tools currently play in shaping perceptions of digital support within CI aftercare. The following sections outline the three thematic dimensions in greater detail.

#### 3.2.2 Motivational drivers

Participants identified time savings, flexibility, and ease of use as the primary motivations for engaging with remote care. Several valued the possibility of managing minor adjustments independently or remaining connected to the clinic after relocation. Overall, remote care was perceived as advantageous in reducing travel burden, increasing scheduling flexibility, and enabling home-based self-monitoring. With regard to dHTP, participants preferred short training sessions of 10–15 min. Bimodally fitted users reported a greater willingness to practice daily, whereas experienced single-sided deafness users preferred short, occasional training. Longer-term or continuous practice periods were generally viewed as unnecessary.

#### 3.2.3 Barriers and facilitators for use

Reported disadvantages of remote care included reduced suitability for older or less digitally experienced users, concerns about diminished personal interaction, and uncertainties regarding technical reliability, stable internet access, and troubleshooting. Despite these concerns, PwCI generally favored a hybrid care model, perceiving digital tools as useful for routine monitoring and self-management while continuing to value in-person appointments for reassurance and direct communication. Most participants indicated that remote-care services should be covered by health insurance and jointly coordinated by manufacturers and CI centers.

Participants varied in their confidence when using digital technologies. Some regarded everyday smartphone and computer use as routine, while others, particularly older or less experienced users, felt uncertain and relied on family support. At the same time, several participants expressed willingness to improve their digital skills if this facilitated remote-care use. Participants reported mixed experiences with dHTP. While some engaged with these applications out of curiosity or as a way to structure at-home practice, others described frustration, monotony, or decreasing motivation over time. Participants emphasized that digital tools should be intuitive, varied, and practically useful in everyday use in order to sustain engagement.

#### 3.2.4 Contextual conditions for engagement

Participants discussed multiple aspects of user experience in dHTP that influence engagement, including content, feedback design, communication channels, and system notifications. These subthemes reflect both usability expectations and broader conditions necessary for sustained remote-care use.

##### 3.2.4.1 Content and usability requirements

Participants described a range of preferences regarding the content of remote care and dHTP. The most frequently mentioned aspect was the opportunity to monitor and evaluate one's own hearing performance, for example, through single-phoneme tasks or reference sentences. Adaptive exercises that adjust to individual progress were perceived as particularly motivating. Music-related training components were also valued, either to enhance listening enjoyment or to improve sound quality perception. Additional suggestions included customizable sound settings (e.g., equalizer adjustments) and practical information such as device-handling tips or links to CI-related news and services.

##### 3.2.4.2 Feedback and motivation

Feedback in remote care was expected to be motivating rather than evaluative. Participants preferred simple confirmations or positive reinforcements emphasizing correct responses instead of numerical scores or detailed performance statistics. Occasional reminders or notifications were regarded as helpful by some but unnecessary for those who described themselves as intrinsically motivated. For dHTP in particular, participants underscored that hearing training is inherently self-directed and depends largely on users’ personal motivation.

##### 3.2.4.3 Warning system

Participants discussed two key functions of a potential warning system in remote care: preventive and diagnostic alerts. While notifications indicating an imminent low battery status were already familiar to most participants, they expressed a clear preference for earlier, more anticipatory warnings that would allow sufficient time for planning and prevent unexpected interruptions. Diagnostic messages indicating possible technical malfunctions were also valued, as they could help differentiate user errors from device-related issues and thereby enhance users’ confidence and autonomy.

##### 3.2.4.4 Communication and contact options

Participants expressed a preference for direct and personal communication channels. Chatbots were largely rejected as impersonal or unhelpful, whereas e-mail and direct in-app chat with familiar clinical professionals were viewed as acceptable alternatives, particularly when enabling fast and uncomplicated contact with the established clinical team. Automated acknowledgments or clearly defined response times were considered helpful for reassurance, while direct professional access remained essential for trust and continuity of care.

### 3.3 Quantitative results

#### 3.3.1 Remote care awareness and preferences

Among the 62 PwCI, 39% (*n* = 24) reported no prior awareness of remote care, while approximately half of the sample reported some level of familiarity without prior use; a small proportion (8%, *n* = 5) had previously used remote-care services. Preferences for in-clinic rehabilitation were highest during initial therapy (68%) and decreased to 50% for follow-up care, while preferences for remote care increased from 16% to 27%. Local hearing-care professionals were selected by 13% during initial and follow-up therapy and by 16% for lifelong aftercare. When asked about responsibility for implementing remote care, 68% assigned this to the CI center, and 16% each to local hearing-care professionals or provided no response.

#### 3.3.2 Motivational patterns in remote CI aftercare (RQ2)

##### 3.3.2.1 ACTA motivation subscales

Analysis of the ACTA (Autonomy and Competence in Technology Adoption) subscales revealed distinct motivational patterns. Self-determined motivation did not significantly deviate from normal distribution (W = 0.97, *p* = .150), whereas externally controlled motivation (W = 0.96, *p* = .026) and perceived competence (W = 0.93, *p* = .002) showed significant deviations. On the five-point scale, mean values were:

- Self-determined motivation: M = 3.43 (SD = 0.76; range = 1–5)
- Externally controlled motivation: M = 1.97 (SD = 0.54; range = 1–3.67)
- Perceived competence (two items) for remote care showed a mean value of M = 3.52 (SD = 0.81; range = 1.5–5).

A paired-samples t-test showed a significant difference between the two subscales, self-determined motivation and externally controlled motivation, t(61) = –12.75, *p* < .001, with a large effect size (Cohen's d = 1.62). Perceived competence showed a moderate positive correlation with self-determined motivation (*ρ* = .60, *p* < .001, one-tailed) and a weak, non-significant negative association with externally controlled motivation (*ρ* = −.21, *p* = .096, two-tailed). Interest in remote care (M = 3.52, SD = 0.99) also correlated with perceived competence (*ρ* = .62, *p* < .001).

Item-level analysis showed higher mean scores for items of the self-determined motivation and competence subscales, and lower mean scores for externally controlled motivation. Item 3 (“…because it helps me with something important”) showed the highest mean score, while Item 6 (“…because it makes a good impression on others”) showed the lowest.

#### 3.3.3 Relationships between perceived competence, digital health literacy, technology affinity, and motivation (RQ3)

##### 3.3.3.1 Digital health literacy (eHEALS) and technology affinity (ATI)

Digital health literacy deviated significantly from normal distribution (W = 0.93, *p* = .002) with a mean of M = 3.94 (SD = 0.73, range = 1.88–5). Technology affinity showed no significant deviation (W = 0.97, *p* = .135) and a mean of M = 4.08 (SD = 0.9, range = 2.44–6).

##### 3.3.3.2 RQ3.1: Correlations between competence, digital health literacy, and technology affinity

Perceived competence for remote care correlated moderately with digital health literacy (*ρ* = .43, *p* < .001, one-tailed).

##### 3.3.3.3 RQ3.2: Associations between self-determined and externally controlled motivational subscales and competence

Perceived competence correlated positively with self-determined motivation (*ρ* = .60, *p* < .001) and showed a weak negative association with externally controlled motivation (*ρ* = –.21, *p* = .096).

##### 3.3.3.4 RQ3.3: Predictors of perceived competence for remote care

A multiple linear regression model significantly explained 42% of the variance in competence [F(4, 57) = 10.32, *p* < .001]. Model diagnostics did not indicate multicollinearity (variance inflation factor < threshold), and residuals were normally distributed (W = 0.97, *p* = .149). Technology affinity was a significant positive predictor (*β* = 0.08, *p* < .001), while age showed a significant negative effect (*β* = −0.03, *p* = .017). Digital health literacy (*β* = 0.06, *p* = .0599) and gender (*β* = −0.18, *p* = .631) were not significant predictors.

## 4 Discussion

This exploratory-sequential mixed-methods study examined how PwCI understand and evaluate remote care, which motivational mechanisms shape their engagement, and how digital competencies influence perceived readiness for remote CI aftercare.

Across qualitative and quantitative findings, a coherent pattern emerged: autonomy and perceived competence as central constructs within Self-Determination Theory (SDT) play a decisive role in motivating PwCI to engage with and express readiness for remote-care solutions, while the need for relatedness and professional reassurance continues to anchor patients’ preference for hybrid models.

### 4.1 Motivational drivers, barriers, and contextual conditions for remote CI aftercare (RQ1)

PwCI perceived remote care predominantly as a meaningful complement to, rather than a substitute for, traditional follow-up care. This preference strongly echoes prior teleaudiology findings demonstrating high satisfaction and feasibility, but sustained reliance on in-person interaction for reassurance (5, 11, 12, 20, 21, 32). The hybrid preference is consistent with broader eHealth literature, indicating that patients' expected acceptance is highest when digital tools enhance, without displacing, established care structures (15).

From an SDT perspective, participants valued the autonomy afforded by remote access, self-monitoring, and flexible scheduling, yet consistently emphasized the need for relatedness and reliable contact with the clinical team. The expectation that responsibility should be shared carefully between CI manufacturers and CI centers reflects users’ preference for structured, guided digital care rather than self-directed management.

Motivational drivers such as convenience, reduced travel burden, and improved scheduling flexibility align with earlier research suggesting that logistical and autonomy-supportive features increase willingness to engage in telehealth (5, 12). The qualitative findings also reinforce that hearing training via dHTP is inherently self-directed and thus particularly dependent on users’ personal motivation and competence. PwCI preferred short, structured, individually relevant exercises, consistent with user-experience principles that show adaptive, varying tasks and competence-enhancing feedback increase engagement (43).

Perceived barriers in this study, namely technical uncertainty, fears of reduced interpersonal interaction, and variable digital proficiency, mirror established challenges in teleaudiology and digital rehabilitation (19, 44). Some participants relied on family support for troubleshooting, highlighting the social dimension of digital readiness. D’Onofrio et al. (19) propose that formally assessing PwCI's digital competence could help clinicians “meet the patient at their comfort level and tailor online interventions accordingly,” highlighting the importance of aligning digital support with users’ individual readiness. This finding aligns with Madanian et al.'s (44) narrative review showing that “patient empowerment, self-management, and personalization” facilitate digital engagement, while “digital literacy, health literacy, and privacy concerns” remain central barriers. Nevertheless, many expressed a willingness to improve their digital skills if remote care offered clear value, demonstrating a relevant readiness for guided onboarding. This suggests that structured support infrastructures facilitating digital onboarding and sustained use may be critical for successful implementation and should be considered within future care models and reimbursement frameworks.

Participants’ expectations regarding usability provide further insight into acceptance conditions. PwCI emphasized intuitive design, adaptive content, positively framed feedback, and transparent communication channels. The desire for real-time system warnings, especially regarding battery status or technical malfunction, indicates that competence-supportive features may help offset users’ uncertainty and reinforce self-efficacy. These preferences correspond with findings that competence-enhancing design elements, such as self-monitoring and timely feedback, support intrinsic motivation and sustained health behavior engagement (15, 43).

Overall, RQ1 shows that remote care is most acceptable when it strengthens autonomy and competence without compromising relatedness, a pattern that aligns closely with SDT and broader digital-health research.

### 4.2 Motivational patterns in remote CI aftercare (RQ2)

Our quantitative ACTA results showed a clear predominance of self-determined motivation over externally controlled motivation. PwCI indicated that the primary reason for using remote care is that it “helps with something important”, underscoring the critical role of personal relevance as a driver of internalization. This aligns with evidence that autonomous motivation predicts sustained engagement and positive outcomes in health behavior change (26, 27, 45). Externally regulated motivation played only a minor role among the PwCI in this study.

Consistent with previous findings, practical benefits such as reduced travel burden and time savings (5), as well as improved compatibility with work and family obligations (12), were perceived as facilitators. However, these factors functioned primarily as situational conveniences rather than as stable external incentives for engagement. Ravichandran et al. (2) similarly reported that sustained engagement with digital health interventions depends less on convenience alone and more on content-related features, including meaningful feedback, self-monitoring opportunities, and supportive interaction mechanisms. In our study, participants likewise highlighted feedback, self-monitoring functions, social support features, and reminders as key facilitators. These elements align with the design-based motivational mechanisms described by Villalobos-Zúñiga and Cherubini (43), which directly support competence (e.g., through feedback and self-monitoring), autonomy (e.g., through optional reminders), and relatedness (e.g., through social support channels).

Notably, PwCI in this study expressed no interest in extrinsic reward structures, such as gamification elements or point-collecting systems. This aligns with Villalobos-Zúñiga and Cherubinís proposition (43) that reward systems may undermine intrinsic motivation by shifting users’ attention away from personal relevance toward external goals. In previous work, these principles were operationalized in the context of an adaptive digital hearing training program, demonstrating how performance-based task adjustment, transparent feedback, and self-monitoring can be technically implemented within CI rehabilitation (8).

Overall, RQ2 indicates that remote-care uptake depends largely on self-determined motivation supported by autonomy and competence, whereas externally controlled motives contribute minimally to engagement.

### 4.3 Competence, digital readiness, and motivation (RQ3)

Across the quantitative results, perceived competence emerged as the strongest psychological correlate of self-determined motivation, demonstrating a robust link between feeling capable and being intrinsically motivated to use remote-care tools. This is highly consistent with SDT, which posits competence as a basic psychological need central to sustained engagement (25). The positive correlation between competence and interest in remote care further underscores that competence is not only a predictor of motivation, but also a key determinant of readiness for adoption (15, 45).

Digital health literacy and technology affinity further contributed to this dynamic. Both constructs showed moderate positive associations with perceived competence, indicating that users with stronger digital skills and greater openness to technology are more confident in performing remote-care tasks. Technology affinity emerged as the most robust predictor in the regression model, whereas digital health literacy fell slightly below statistical significance; this pattern may reflect sample size constraints and heterogeneity in digital proficiency. Nevertheless, the non-normal distribution of digital health literacy in the sample underscores meaningful variability in digital readiness, mirroring population-level disparities reported in German cohort studies (41). Such heterogeneity suggests that remote-care interventions risk amplifying digital inequities unless targeted support (infra-)structures are implemented and reimbursed.

The literature corroborates this interpretation. Systematic reviews consistently identify low digital literacy, insufficient training, and perceived technical complexity as key barriers to digital health adoption in hearing rehabilitation and chronic disease management (2, 44, 45). Conversely, studies highlight that initiatives fostering digital skill-building increase engagement and adherence, particularly when they enhance users’ confidence and self-efficacy (15). Hepburn et al. (15) highlight that effective and equitable digital health strategies require addressing infrastructural and literacy barriers, actively engaging health care providers through training and co-design, and ensuring multistakeholder involvement. These findings support our conclusion that competence-enhancing design and onboarding strategies are pivotal for remote-care uptake. Finally, emerging evidence suggests that digital health literacy may act as a mediator through which health information is translated into behavior (46). In this study, higher literacy appeared to facilitate the interpretation of remote-care tasks as meaningful and manageable, thereby supporting competence and motivation.

dHTP appear to play a special role in this context, functioning as a low-threshold entry point into remote CI aftercare. By providing structured, home-based tasks requiring repeated digital interaction, dHTP are regarded to foster both digital health literacy and self-directed learning behaviors. However, their ability to promote sustained engagement depends heavily on design characteristics, namely adaptive content, intuitive interaction flows, and positive, competence-supportive feedback, consistent with reported evidence (3, 6). When aligned with users’ daily listening needs, dHTP may not only strengthen motivation but also serve as preparatory scaffolding for broader remote-care participation.

Taken together, RQ3 demonstrates that digital readiness and user experience collectively shape perceived competence, which in turn drives autonomous motivation and readiness for remote-care engagement.

### 4.4 Integrated interpretation (mixed-methods meta-inferences)

The integration of qualitative and quantitative findings provides a consolidated explanatory model for how PwCI engage with remote CI aftercare. This integration reflects the exploratory-sequential design, in which qualitative findings informed the interpretation of quantitative results. Across methodological strands, a coherent pattern emerged in which perceived competence functions as the central psychological link between digital readiness, motivational regulation, and actual engagement. Taken together, these findings converge on a multilevel interpretation (see Figure 1):

- Autonomy-supportive components increase willingness to engage.
- Competence-supportive components determine whether engagement can be sustained.
- Relatedness-supportive structures ensure that digital care remains trustworthy and embedded within the clinical relationship.
- Perceived competence emerges across analyses as a central factor associated with how digital literacy, technology affinity, and motivational processes translate into actual behavior.
- dHTP function as both motivational and skill-building instruments, preparing users for broader remote-care tasks by offering guided practice in digital self-management.

This meta-inference aligns with telehealth research emphasizing personalization, empowerment, and structured support pathways (19, 30, 44). Beyond this existing work, the present study adds evidence from a mixed-methods investigation in PwCI showing that perceived competence for remote CI aftercare may be understood as a key enabling condition linking digital skills and motivational processes.

### 4.5 Limitations

This study has several limitations. First, the use of remote care was considered hypothetical by most PwCI, potentially leading to uncertainties in their responses. The findings reflect perceived competence, motivation, and readiness for engagement rather than actual usage behavior or clinical outcomes. Future evaluations during actual use are needed to address this. Self-assessments of digital competence may be influenced by social desirability bias. Moreover, the web-based study format may have resulted in selection bias, potentially favoring participants with higher levels of digital affinity. Offering alternative formats could help achieve a broader and more representative sample. The quantitative sample reflects recruitment within a defined three-month period in a single tertiary CI center, which may limit the generalizability of the findings. Future studies with extended recruitment periods and multi-center designs may allow for larger samples and broader representation. In addition, the small sample size in the qualitative phase limits the generalizability of these findings. The qualitative results should therefore be interpreted as reflecting a range of experiences within the studied cohort rather than being broadly generalizable.

Finally, because a validated German version of the ACTA scale was not available, the translated instrument may have introduced measurement uncertainty despite careful adaptation.

The findings of this study should be interpreted within the specific healthcare context in which the data were collected. Variations in healthcare systems, access to digital technologies, geographical access conditions and cultural attitudes toward digital health may influence patients’ engagement with remote CI aftercare and thereby limit the transferability of the findings. Accordingly, the generalizability of the results to other settings may be constrained, and further research across diverse cultural and healthcare contexts is warranted.

### 4.6 Future directions and design considerations

Our findings offer several implications for advancing the design and implementation of remote CI aftercare. As self-determined motivation and perceived competence were central to engagement, future remote-care systems need to incorporate design features that support autonomy (e.g., flexible task timing, meaningful self-monitoring), competence (e.g., intuitive interfaces, adaptive feedback, guided onboarding), and relatedness (e.g., clear communication channels and integration with the clinical team).

Given the observed variability in digital readiness, targeted training to strengthen digital health literacy and technology affinity should be considered an integral component of remote-care implementation. Such interventions may help reduce disparities and empower PwCI who are less confident in navigating digital tools. Hybrid models that combine digital and in-person follow-up appear particularly promising, offering flexibility while preserving the relational aspects of care that many users value.

Social support also emerged as a relevant facilitator. Future remote-care concepts may benefit from including optional pathways for family or caregiver involvement, particularly for older PwCI, parents of children with CI, or those with limited digital competence. Additionally, iterative co-design processes involving PwCI and clinicians may enhance usability, ensure clinical relevance, and align digital workflows with users’ daily listening needs.

From a theoretical perspective, the present findings extend the application of Self-Determination Theory (SDT) in the context of digital health and CI aftercare. The results suggest that perceived competence represents a central factor associated with engagement and highlight the interplay between motivational regulation and digital readiness.

Rather than demonstrating causal relationships, the findings support an SDT-informed framework for understanding how psychological and technological factors jointly shape engagement with remote care. In this context, perceived competence may be understood as a key prerequisite for effective engagement with digital rehabilitation tools and their integration into daily routines.

### 4.7 Conclusion

Our study demonstrates that the successful adoption of remote CI aftercare depends on the interplay between digital readiness, motivational regulation, and perceived competence. While autonomy-supportive features such as flexible access and self-monitoring may increase initial interest, sustained engagement requires competence-supportive design elements and reliable professional connectedness. Across qualitative and quantitative findings, perceived competence emerged as the central psychological factor linking digital readiness with self-determined motivation and readiness to engage with remote-care tools.

These findings suggest that remote-care concepts for PwCI should prioritize competence-supportive system design, targeted user onboarding, and integration within hybrid care pathways to ensure and improve accessibility, adherence, and long-term rehabilitation benefit and outcome.

## Data Availability

The raw data supporting the conclusions of this article will be made available by the authors, without undue reservation.
