# Supplementary material for: The role of perceived competence in remote cochlear implant aftercare: a mixed-methods study
Source: Front Digit Health. 2026 Jun 3;8:1803067. doi: 10.3389/fdgth.2026.1803067 (PMC13271927; doi:10.3389/fdgth.2026.1803067)
Supplement: Supplementary file 1 [file Table1.pdf]

Supplementary Material

Main categories, subcategories, number of coded segments and representative quotations (3 group interviews, n = 9)

Main Category	Subcategory	N	Representative Quotation	Participant
Remote care	Advantages	22	"Remote care saves me hours of travel time and allows me to manage appointments around my work."	SSD_03, SSD group, Interview 1
	Disadvantages	25	"I can't imagine how older people would manage this on their own—it's too technical and they would miss the personal contact."	bm_03, bimodal group, Interview 2
	Personal preferences		"Remote care should come from the manufacturer, but the clinic has to keep an eye on things and be available if something goes wrong."	SSD_02, SSD group, Interview 1
	Financing	9	"In the end, the health insurance should pay for it—it's part of aftercare."	bm_03, bimodal group, Interview 2
Technology affinity	Practical Handling and Digital Competence	40	"I can use my phone for the basics, but when something doesn't work, I usually ask my daughter for help. Still, I'd like to learn how to do more on my own."	Bl_02, bilateral group, Interview 3
	Prior Experience with dHTPs	22	"Using a hearing training app feels different from normal apps; you have to concentrate much more."	Bl_01, bilateral group, Interview 3
Motivation	Personal motives	8	"I would just try it out of curiosity, to see how it works."	Bm_03, bimodal group, Interview 2
	Usage period	16	"No one trains like that for a whole year. At some point, you just lose motivation."	SSD_02, SSD group, Interview 1
User Experience /Content	Content and Usability Requirements	35	"Only now with the implant do I notice how many different 'sh' and 'ch' sounds there are. It would be helpful if the training adapted to such weak points."	Bm_03, bimodal group, Interview 2
	Feedback and Motivation	9	"It should be motivating — even if I make mistakes, the focus should be on what I got right rather than what went wrong."	Bm_02, bimodal group, Interview 2
	Notification	4	"If I really want to learn, I'll do it myself — I don't need a reminder."	SSD_03, SSD group, Interview 1
	Systems – Warning system	28	A [battery charge] reminder would be useful — but it has to come on time, since you don't always check the battery level in everyday life."	SSD_01, SSD group, Interview 1

	Communication and Contact options	24	“A direct contact would simply be easier — that’s what I would prefer.”	SSD_01, SSD group, Interview 1

Note: Original quotes were in German and translated into English; some were abbreviated for clarity. dHTP = digital hearing training programs; SSD = single sided deafness;
